# Effect of Exercise on Photoperiod-Regulated Hypothalamic Gene Expression and Peripheral Hormones in the Seasonal Dwarf Hamster *Phodopus sungorus*

**DOI:** 10.1371/journal.pone.0090253

**Published:** 2014-03-06

**Authors:** Ines Petri, Rebecca Dumbell, Frank Scherbarth, Stephan Steinlechner, Perry Barrett

**Affiliations:** 1 Department of Zoology, University of Veterinary Medicine, Hannover, Germany; 2 Rowett Institute for Nutrition and Health, University of Aberdeen, Aberdeen, United Kingdom; University of Texas Southwestern Medical Center, United States of America

## Abstract

The Siberian hamster (*Phodopus sungorus*) is a seasonal mammal responding to the annual cycle in photoperiod with anticipatory physiological adaptations. This includes a reduction in food intake and body weight during the autumn in anticipation of seasonally reduced food availability. In the laboratory, short-day induction of body weight loss can be reversed or prevented by voluntary exercise undertaken when a running wheel is introduced into the home cage. The mechanism by which exercise prevents or reverses body weight reduction is unknown, but one hypothesis is a reversal of short-day photoperiod induced gene expression changes in the hypothalamus that underpin body weight regulation. Alternatively, we postulate an exercise-related anabolic effect involving the growth hormone axis. To test these hypotheses we established photoperiod-running wheel experiments of 8 to 16 weeks duration assessing body weight, food intake, organ mass, lean and fat mass by magnetic resonance, circulating hormones FGF21 and insulin and hypothalamic gene expression. In response to running wheel activity, short-day housed hamsters increased body weight. Compared to short-day housed sedentary hamsters the body weight increase was accompanied by higher food intake, maintenance of tissue mass of key organs such as the liver, maintenance of lean and fat mass and hormonal profiles indicative of long day housed hamsters but there was no overall reversal of hypothalamic gene expression regulated by photoperiod. Therefore the mechanism by which activity induces body weight gain is likely to act largely independently of photoperiod regulated gene expression in the hypothalamus.

## Introduction

The Siberian hamster, *Phodopus sungorus* (also known as the Djungarian hamster), is an exemplar of responsiveness to seasonal photoperiod. As summer turns to autumn, the Siberian hamster experiences physiological, morphological and behavioural adaptations including gonadal involution, change in pelage colour, a large reduction in body weight, reduction in food intake and, by early winter, induction of torpor. These responses are in anticipation of reduced food availability and a decrease in the ambient temperature of this species' natural environment [1]. The neural mechanisms underlying these physiological and associated behavioural changes are not yet understood, but several investigators have identified a significant number of photoperiod regulated genes, particularly in the hypothalamic region, which may underpin the physiological and behavioural responses [2]–[8].

The Siberian hamster can lose up to 40% of body weight over a 12–16 week interval when transferred from a long day photoperiod (LD; 16 h light∶8 h dark) to a short day photoperiod (SD; 8 h light∶16 h dark), but this can be reversed by switching hamsters back into LD or occurs in extended periods of SD, also known as the photorefractory response [9]–[13]. This model of reversible body weight regulation offers opportunities to identify new components or structures in the brain which are involved in the regulation of body weight.

A number of studies have looked at expression of genes for the principal neuropeptides involved in the homeostatic mechanism of appetite and energy balance (*Npy*, *Pomc*, *Agrp* and *Cart*) in the arcuate nucleus (ARC) of the hypothalamus. These investigations have concluded that neuropeptides belonging to a homeostatic mechanism of body weight and food intake regulation may not be significantly involved in the seasonal reduction of body weight and food intake of the Siberian hamster [14], [15], although there may be some scope for a seasonal change in prohormone processing of POMC [16]. Consequently other genes are likely to underpin the mechanism of autumnal weight loss and reduction in food intake. In this regard, we and others have identified a significant number of genes changing in response to a change in photoperiod from LD to SD including, for example, down-regulation of histamine H3 receptor, increased expression of VGF and melanocortin 3 receptor (MC3R) in the dmpARC [2], [17], down regulation of suppressor of cytokine signalling 3 (SOCS3) and MC3R and increased expression of the leptin receptor ObRb in the ARC [18], [19]. Underpinning all these changes in gene expression in the hypothalamus, the reduction in body weight, food intake and the down-regulation of the reproductive system is a photoperiod-dependent reduction of thyroid hormone availability to the hypothalamus [4].

The reduction in thyroid hormone availability is brought about by an increased expression of type III deiodinase (Dio3), which metabolises active thyroid hormone T3 to inactive derivatives. An associated reduction in the expression of type II deiodinase (Dio2), responsible for the synthesis of T3 from the precursor T4, may also occur, contributing to reduced thyroid hormone availability [4], [8], [20], [21]. The deiodinase enzymes are expressed in tanycytes, cells which line the hypothalamic ventricular region [22], where a number of other photoperiod dependent changes in gene expression have been found to occur [21], [23], [24] and are likely to be involved in seasonal physiological responses.

In the periphery, insulin circulates at lower concentrations in SD Siberian hamsters in comparison to their LD counterparts [25]. This reduction in peripheral insulin may be indicative of increased peripheral insulin sensitivity in SD and therefore a more insulin resistant state in the heavier LD phenotype. Furthermore, insulin has recently been implicated in playing a role in lipogenesis in the Siberian hamster [26]. Circulating serum fibroblast growth factor 21 (FGF21) concentrations in Siberian hamsters have been found to differ between LD and SD [27]. FGF21 is produced by several tissues in the body, including adipose tissue, liver and muscle, and is implicated in control of energy metabolism, lipolysis, and feeding behaviour (reviewed in e.g. [28]).

Remarkably, a number of physiological responses to SD can be significantly blunted or reversed when Siberian hamsters are given access to a running wheel [29]–[31]. The impact of running wheel activity on seasonal physiology is most overtly seen in the response of body weight. When given free access to a running wheel at the time of switch to SD, the hamsters do not show the typical weight loss. If progress toward weight loss has already occurred, the hamsters will gain weight. Such weight increase can be reversed by preventing running wheel activity [31]. Furthermore, in a natural photoperiod environment, access to a running wheel at the inflexion point (nadir) of the body weight cycle, significantly enhances spring trajectory of body weight increase and enhances testicular recrudescence [30].

Recently it has been reported that hamsters kept in SD may not only reduce fat mass, but also reduce the amount of lean mass, including reduced organ size. It is also reported that access to a running wheel induces growth demonstrated by the femora length and increase in organ sizes [30], [31]. We would therefore suggest that the Siberian hamster represents a model of disrupted or reversible growth. Our hypothesis for this study is that running wheel activity reverses the photoperiod driven changes in gene expression and peripheral circulating hormones that underpin the physiological response of decreased body weight, food intake, and growth. We have therefore analysed some of the key changes in photoperiod driven gene expression to determine whether running wheel activity reverses SD induced photoperiod changes in gene expression and peripheral hormones.

## Materials and Methods

### Ethics statement

All experiments were in accordance with the German Animal Welfare Act and all animal experiments in our laboratory were approved by or registered with the Animal Welfare Service at LAVES (Lower Saxony State Office for Consumer Protection and Food Safety) by notice of 16-02-2010.

Male Siberian hamsters were bred and raised under a natural photoperiod (Hannover, Germany; 52°N latitude) and natural ambient temperatures before the summer solstice. They were transferred to artificial LD 16∶8 after weaning and were at least 3 months old (i.e. adult) at the beginning of the experiments. Food (hamster breeding diet, Altromin 7014, Lage, Germany) and water was available *ad libitum*, supplemented weekly by a slice of apple. Experimental hamsters were singly housed indoors at an ambient temperature of 21±1°C, starting four weeks after weaning and throughout all experiments. At the end of the experiments, hamsters were humanely culled using carbon dioxide followed by cervical dislocation.

### Animals and tissue collection

A total of five experiments were conducted using weight-matched adult male hamsters. The experiments lasted either 8 (two experiments), 12 (two experiments) or 16 weeks during which time running wheel access was provided. Hamsters were housed in either long-day (LD; 16 h light∶8 h dark) or short-day (SD; 8 h light∶16 h dark) photoperiod. Overhead lighting was provided by fluorescent tubes (Lumilux LF11, Osram, Germany) resulting in a light intensity of ca. 200–350 Lux at cage level. During the dark phase, illumination was limited to dim red light of <5 Lux (Osram, Darkroom red, 15 W). In each experiment hamsters were divided into four groups, two LD and two SD. One group of hamsters in LD (LD-RW) and one group in SD (SD-RW) received a running wheel (RW; Ø 14.5 cm). The remaining hamsters represented sedentary LD and SD control groups (LD-C and SD-C respectively). The sample size was 6–8 animals per group unless otherwise stated. Accumulated food intake was determined over the course of a 12 week experiment. Animals were culled with carbon dioxide at the end of all experiments, 3–4 h after lights on. In the 8 and 12 week experiments, brains were immediately dissected, frozen on dry ice and stored at −80°C for later *in situ* hybridization. Organs (liver, kidneys, heart, testes and right epididymal fat pads) were dissected, weighed and frozen. Decapitated carcasses of one 8 week and the 16 week experiment were stored at −80°C until used for measurement of body composition. To assess fat and lean mass composition, each carcass was placed in a sealed plastic bag, heated to 37°C in a waterbath and subsequently scanned by nuclear magnetic resonance imaging (MRI) (Echo MRI ™, Whole Body Composition Analyser, Echo Medical Systems, Houston, Texas).

Liver glycogen content was determined by measuring glucose after enzymatic breakdown of glycogen [32]. Lipids were analysed by gas chromatography after conversion to the fatty acid methyl esters [33].

In this study, the focus of gene expression analysis was the 8 week experiments since a primary goal was to determine an effect on Dio3 gene expression which peaks at about 8 weeks into SD photoperiod before declining [4]. Where stated, analysis of gene expression was performed on brains of hamsters of the 12 week experiment to compare mRNA expression of a limited number of genes determined worthy of follow up at this later time point. Serum from a second 12 week experiment was prepared for the analysis of circulating concentrations of insulin and FGF21. Only body weight, organ weight and body composition was assessed at 16 weeks to compare the effect of long term running wheel activity, particularly in relation to fat mass. A summary of the measurements made in each of the experiments is presented in Table 1.

**Table 1:** A summary of length of photoperiod treatment and the measurements/analysis done within these experiments.

	Exposure to short photoperiod (with or without running wheel)		
	8 weeks	12 weeks	16 weeks
Body composition	X	-	X
Food intake	-	X	-
Serum concentrations			
Insulin	-	X	-
FGF21	-	X	-
Organs			
Weights of testes, EWAT, heart, kidney and liver	x	-	x
Liver glycogen and lipids	x	-	-
Hypothalamic expression of mRNA			
AgRP	x	-	-
NPY	X	X	-
POMC	X	X	-
CART	X	X	-
SRIF	X	X	-
Dio2/Dio3	X	-	-
TRH	X	X	-
MCT-8	X	-	-
Vimentin	X	-	-
GPR50	X	-	-
VGF	X	-	-

### Riboprobes

Riboprobes complementary to fragments of the required DNA sequences were generated from Siberian hamster, mouse or rat brain cDNAs by RT-PCR as described previously ([2], [4], [21], [25], [34]–[38] and Table S1). Templates for riboprobe synthesis were generated by PCR amplification of the insert from plasmid DNA with M13 forward and reverse primers which span both insert and polymerase transcription binding and initiation sites in the host vectors. One hundred micrograms of PCR product were used in an *in vitro* transcription reaction with T7, T3 or SP6 polymerases as appropriate in the presence of ^35^S-uridine 5-triphosphate (Perkin-Elmer, Buckinghamshire, UK) for radioactive *in situ* hybridization.

### *In situ* hybridization

Coronal sections (14 µm) of the hypothalamus were collected for the ARC and PVN region, respectively. *In situ* hybridizations were carried out as described previously [39].

Briefly, frozen slides were fixed in 4% PFA in 0.1 m PBS, and acetylated in 0.25% acetic anhydride in 0.1 m TEA, pH 8. Radioactive probes (approximately 10^6^ cpm) were applied to the slides in 70 µl hybridization buffer containing 0.3 M NaCl, 10 mM Tris-HCl (pH 8), 1 mM EDTA, 0.05% tRNA, 10 mM DTT, 0.02% Ficoll, 0.02% polyvinylpyrrolidone, 0.02% BSA and 10% dextran sulfate. Hybridization was performed overnight at 58°C. Following hybridization, slides were washed in 4× SSC (1× SSC is 0.15 M NaCl, 15 mM sodium citrate), then treated with ribonuclease A (20 µg/µl) at 37°C and finally washed in 0.1× SSC at 60°C. Slides were dried and apposed to autoradiographic Biomax MR film (Kodak, Rochester, New York) for several hours to days.

### Image analysis

Films were scanned at 600 dpi. Quantification was carried out using Image J 1.37v software (Wayne Rasband, National Institutes of Health, USA). For each probe, three sections spanning a selected region of the hypothalamus were chosen for image analysis. Integrated optical density for each selected region was obtained by reference to a standard curve generated from the autoradiographic ^14^C microscale (Amersham) with background levels set zero. An average (± SEM) for the integrated optical densities for all sections of one animal and for all animals in one group was calculated.

### Serum hormone concentration determination

Terminal blood samples were collected into a microfuge tube and held on ice until all samples had been collected. After all samples were collected, tubes were spun at 4°C for 15 min at 1000 g. Serum was removed to new microfuge tubes and stored until required. Non-fasted levels of Insulin and FGF-21 concentrations were measured in serum samples from one 12 week experiment.

ELISAs were performed on thawed serum according to manufacturers' instructions. Insulin concentration was measured using a rat insulin ELISA kit (Mercodia, Uppsala, Sweden), and FGF-21 was measured using a rat/mouse ELISA kit (Millipore, MA, USA), following instructions for mouse samples. Each sample was measured in duplicate within one assay. The intra assay coefficient of variation for these assays were: Insulin assay 8.5% and FGF21 assay 6.4%.

### Statistical analysis

Statistical analysis was performed in Sigmaplot v12 (Jandel). The statistical test applied in this study was two-way ANOVA with photoperiod and activity as factors. If appropriate, data was log_e_ transformed prior to test. Differences between groups were revealed with Tukey post-hoc test for multiple comparisons as appropriate. Where normality or variance tests failed, non-parametric analysis was conducted within the statistical software R using the Kruskal-Wallis test; post-hoc tests were performed by using the R-function kruskalmc, which implements the method detailed in Siegel and Castellan [40]. A two-tailed t-test was used to compare *Dio3* mRNA expression within SD groups. For the insulin data measurements were bounded from above, i.e. for large concentrations only the upper bound could be observed instead of the actual value. For this reason we used Cox regression to test for differences between the groups, a non-parametric method widely used in survival analysis, where such right-censored data are common. A Bonferroni correction was used to adjust for multiple testing. Values are expressed as mean+SEM and differences were considered significant if P<0.05.

## Results

### Body mass

Body mass data are shown for one 8 week and the 16 week experiment, with similar weight loss profile achieved in the other 8 and 12 weeks experiments. As the body weight profiles are similar for the 8 week experiments we have used brain sections from both groups to derive the overall picture of gene expression patterns in response to photoperiod-running wheel activity treatment.

**Figure 1:**
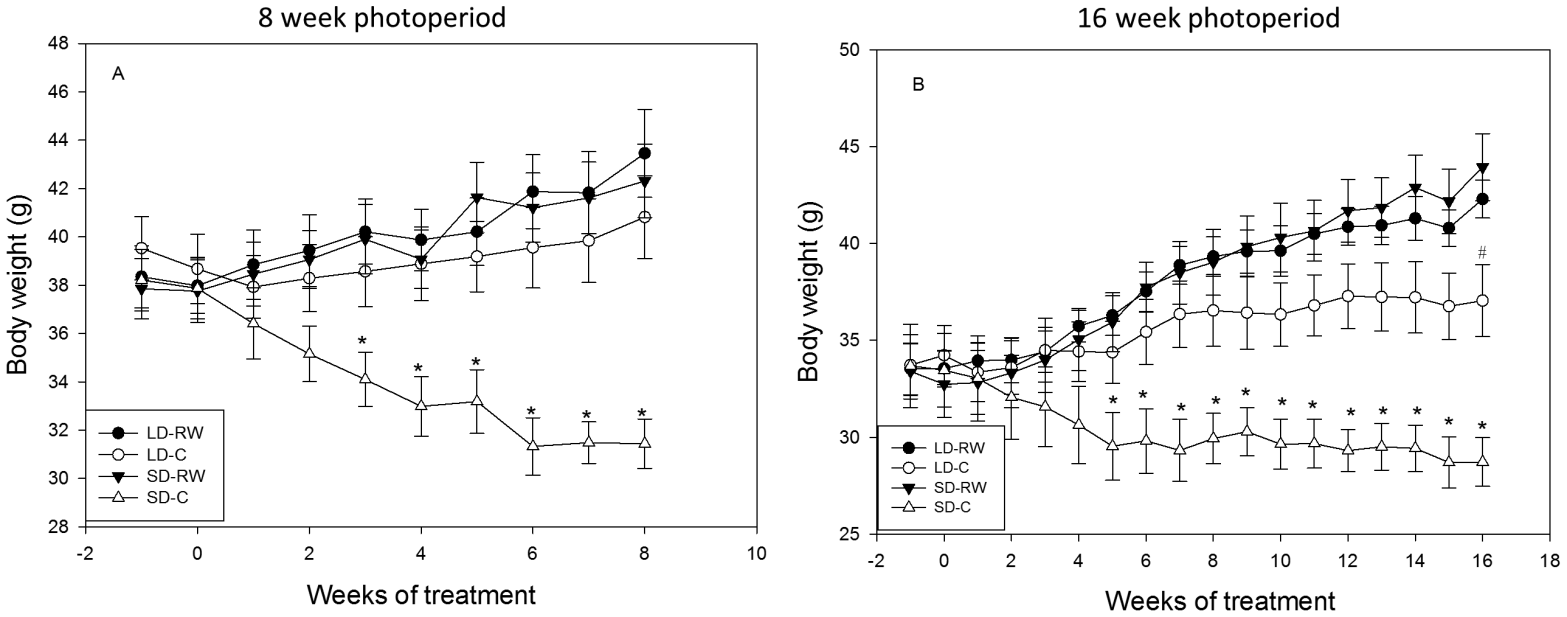
The effect of running wheel activity on body weight. A) Mean body mass (g) of adult male Siberian hamsters during an 8 week exposure to short day (SD) or long day (LD) photoperiod with or without access to a running wheel (n = 7 per group). * SD-C significantly different vs SD-RW (P<0.05 or lower). B) 16 week photoperiod exposure (n = 6–9 per group) ^a^ SD-C significantly different to the other 3 groups (P<0.05 or lower). # Significantly different from LD-RW group (P<0.05).

In the 8 week experiment, body weight in the sedentary SD group (SD-C) decreased with time and was significantly different from all other groups from week 3 onwards. After 8 weeks there was an effect of photoperiod, activity and an interaction between photoperiod and activity on body mass (two-way ANOVA; F(1,24) = 11.93, P = 0.002, F(1,24) = 19.94; P<0.001, F(1,24)7.29, P = 0.013 respectively). Post-hoc analysis shows this was due to the reduction in SD-C group compared to both LD-C and SD-RW groups (P<0.001, Figure 1A). In the 16 week experiment (Figure 1B), body weight decreased in the SD-C with time and was significantly different from all other groups by week 5. After 16 weeks there was an overall effect of photoperiod, activity and an interaction between photoperiod and activity on body mass (F(1,27) = 4.272, P = 0.049, F(1,27) = 40.103, P<0.001, F(1,27) = 9.534, P = 0.005 respectively; Figure 1B). In this 16 week experiment, body weight of both RW hamster groups increased at a faster rate than the LD-C group to become significantly different from the LD-RW group in the last week of the study (P = 0.034).

### Food intake

Food intake, measured as accumulated food intake, was determined for the duration of a 12 week experiment. By week 12 there was no effect of photoperiod (F(1,19) = 1.385, P = 0.254), there was an overall effect of running wheel activity (F(1,19) = 18.31, P<0.001), but no interaction (F(1,19) = 1.297 P = 0.269). Post-hoc analysis revealed no difference in food intake between running wheel groups or between sedentary groups, but both running wheel groups had a higher food intake compared to sedentary hamsters (LD-RW 462.1±30.7 g, n = 6, LD-C 390.6±8.8 g, n = 6; SD-RW 461.3±28.1, n = 6 SD-C 353.8±18.0 g, n = 5).

### Body composition

**Figure 2:**
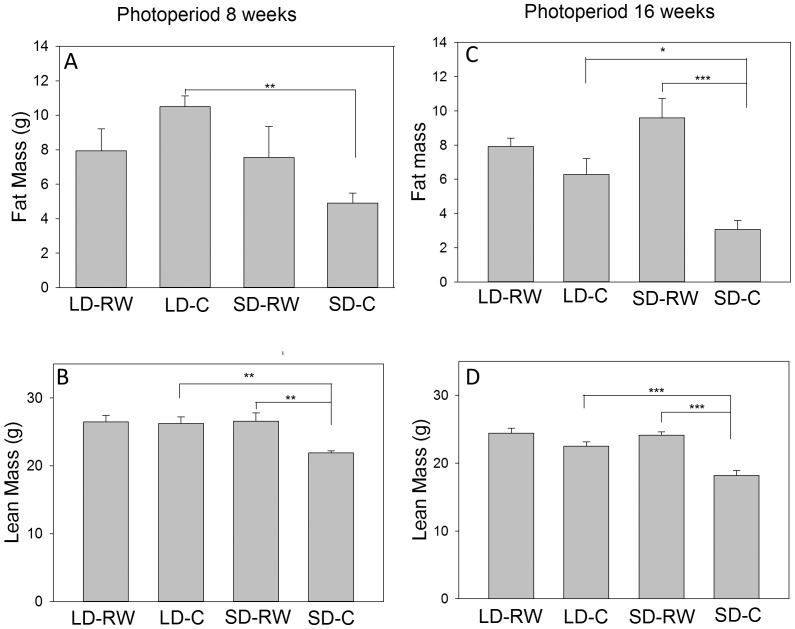
The effect of running wheel activity on fat and lean mass. (A) Fat mass of Siberian hamsters held in photoperiod for 8 weeks; (B) Lean mass of Siberian hamsters held in photoperiod for 8 weeks; (C) Fat mass of Siberian hamsters held in photoperiod for 16 weeks; (D) Lean mass of Siberian hamsters held in photoperiod for 16 weeks (n = 6–9 per group). *P<0.05; **P<0.01; ***P<0.001. LD-RW, long days with running wheel access; LD-C, long days without running wheel access; SD-RW, short days with running wheel access; SD-C, short day without running wheel access.

Lean and fat mass was determined by Nuclear Magnetic Resonance scanning on decapitated hamsters of an 8 week and 16 week experiment. Fat mass or lean mass with body weight are highly correlated at either 8 weeks or 16 weeks of treatment (P<0.001 for all correlations; Figure S1). In absolute mass by 8 weeks in SD, there was an overall effect of photoperiod to reduce fat mass (LD-RW 7.93±1.28 g, LD-C 10.5±0.62 g, SD-RW 7.54±1.82 g, SD-C 4.91±0.57 g; F(1,20) = 6.356; P = 0.02), no effect of activity (F(1,20)<0.001, P = 0.980), but an interaction between photoperiod and activity (F(1,20) = 4.799, P = 0.04). Since fat mass in LD-RW and SD-RW hamsters were very similar, the photoperiod effect was due to the significantly decreased fat mass (53%) in SD-C animals compared to the LD-C group (P = 0.003, Figure 2A). After 16 weeks of treatment, there was no overall effect of photoperiod (F(1,27) = 0.759, P = 0.391. However, there was an effect of activity (F(1,27) = 21.798, P<0.001) and an interaction between photoperiod and activity (F(1,27) = 7.780, P = 0.01). Compared to LD-C hamsters, the SD-C hamsters reduced fat mass by 51% while the running wheel activity significantly increased fat mass by 53% in the SD-RW group (figure 2C). There was a 26% increase in fat mass in LD-RW hamsters compared to LD-C, but this did not achieve significance (LD-RW 7.92±0.49 g, LD-C 6.27±0.94 g, SD-RW 9.59±1.12 g, SD-C 3.08±0.52 g).

After 8 weeks, lean mass was significantly greater in RW hamsters compared to sedentary hamsters (LD-RW 26.47±0.94 g, LD-C 26.24±0.94 g, SD-RW 26.55±1.25 g, SD-C 21.88±0.33 g; F = 6.934; P = 0.016). There was an overall effect of SD photoperiod to reduce lean mass (F(1,20) = 6.195; P = 0.022), an effect of activity (F(1,20) = 7.595, P = 0.012) and an interaction between photoperiod and activity (F(1,20) = 6.316 P = 0.021). Post-hoc analysis shows there was a 17% reduction in lean mass in the SD-C group compared to SD-RW hamsters (P = 0.002, figure 2B).

In the 16 week experiment, photoperiod had no overall effect (F(1,27) = 0.759, P = 0.391) but there was a small but significant increase in lean mass due to running wheel activity (F(1,27) = 36.94; P<0.001) and an interaction between photoperiod and activity (F(1,27) = 7.78, P = 0.01; LD-RW 24.4±0.74 g, LD-C 22.48±0.64 g, SD-RW 24.13±0.46, SD-C 18.19±0.71 g; Figure 2D). These effects are attributable to a greater lean mass of both RW groups compared to their sedentary controls and a 19%–25% reduction in lean mass of the SD-C group relative to the LD-C and SD-RW groups (P<0.001).

### Organ weights

**Figure 3:**
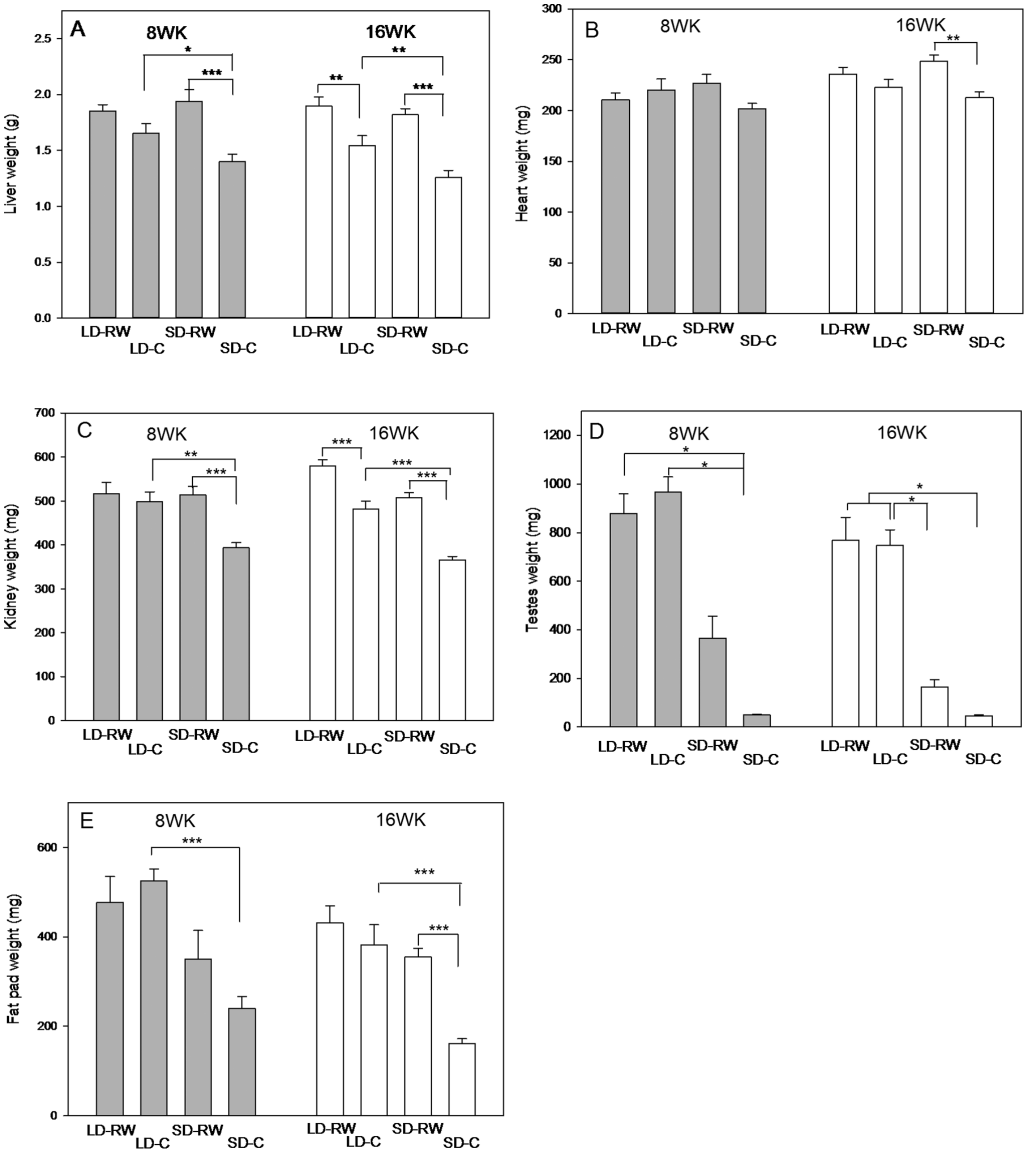
The effect of running wheel activity on organ mass. (A) Liver, (B) heart, (C) kidney, (D) paired testes, (E) right epididymal fat pad of adult male Siberian hamsters after 8 (filled bars) or 16 week (open bars) exposure to long day (LD) or short day (SD) photoperiod with (RW) or without (C) a running wheel (n = 6 per group 8 weeks or 6–9 per group 16 weeks). Relevant significant differences are shown *P<0.05, **P<0.01, ***P<0.001.

Organ weights were determined at the end of an 8WK and 16WK experiment (Figure 3).

### Liver

After 8 weeks of photoperiod/running wheel treatment, there was no overall effect of photoperiod but there was an overall effect of running wheel activity and interaction between both to increase liver mass (F(1,20) = 1.041, P = 0.320, F(1,20) = 1.041, P = 0.32, F(1,20) = 21.49; P<0.001, F(1,20) = 4.582, P = 0.045 respectively) (Figure 3A). Post-hoc analysis revealed the significant interactions at this 8 week time point were liver masses of hamsters in the SD-C group were significantly reduced to both LD-C (P = 0.037) and SD-RW (P<0.001) groups. After 16 weeks of treatment both photoperiod and activity had an effect on liver mass, but no interaction between photoperiod and activity (LD-RW 1.9±0.08 g, LD-C 1.54±0.09 g, SD-RW 1.82±0.05 g, SD-C 1.26±0.06 g; F = 5.91, P = 0.022 and F(1,27) = 37.36, P<0.001, F(1,27) = 1.975, P = 0.171 respectively) Post-hoc analysis show that in addition to a significant difference between SD-C and SD-RW (P<0.001) and LD-C groups (P = 0.008) the increase in liver mass of the LD-RW group compared to LD-C was significant (P = 0.003).

### Heart

Heart mass did not differ between the groups after 8 weeks of treatment (LD-RW 235±7 mg, LD-C 222±8 mg, SD-RW 248±6 mg, SD-C 212±5 mg; Figure 3B) but after 16 weeks there was an overall effect of activity (F(1,27) = 12.38, P = 0.002), but no effect of photoperiod (F(1,27) = 0.037, P = 0.85) or interaction between photoperiod and activity (F(1,27) = 2.646, P = 0.115). Post-hoc analysis shows a small significant increase in heart mass of the SD-RW group compared to SD-C (P = 0.001).

### Kidney

After 8 weeks of treatment there was an effect of photoperiod and activity on kidney mass (LD-RW 516±26 mg, LD-C 499±22 mg, SD-RW 514±20 mg, SD-C 394±11 mg; photoperiod; F(1,20) = 6.83; P = 0.017; activity, F(1,20) = 11.12; P = 0.003) with an interaction between photoperiod and activity (F(1,20) = 6.25, P = 0.021) (Figure 3C). Post-hoc analysis shows the SD-C group has a significantly reduced kidney mass compared to LD-C (P = 0.002) and SD-RW (P<0.001) groups. The overall effect of photoperiod, activity and the interaction between photoperiod and activity were maintained after 16 weeks of treatment (580±10 mg LD-RW, 510±10 mg SD-RW 480±20 mg LD-C vs 370±10 mg; F(1,27) = 49.80, P<0.001 and F(1,27) = 80.76, P<0.001, (F(1,27) = 5.625, P = 0.025, respectively). Post-hoc analysis shows a reduction in kidney mass in the SD-C group compared to the LD-C group (P<0.001). There was a significant increase caused by running wheel activity in LD (P = 0.004) and SD (P<0.001).

### Testes

After 8 weeks there was an effect of photoperiod on paired testis weight (PTW). Non-parametric analysis reveals PTW in the SD-C group to be significantly different (P<0.05) to both LD-C and LD-RW groups. The SD-RW group at a value between the LD groups and the SD-C group was not significantly different to either LD groups or the SD-C group (LD-RW 879±82 mg, LD-C 968±61 mg, SD-RW 365±90 mg, SD-C 49±3 mg). In the 16 week experiment, PTW of both LD-C and LD-RW groups were significantly different from SD-C (P<0.05) and LD-C group significantly different from the SD-RW group. However, as at the 8 week time point at a value between groups, the SD-RW group was not significantly different from the SD-C group or the LD-RW group (LD-RW 768±92 mg, LD-C 747±63 mg, SD-RW 164±28 mg, SD-C 50±2 mg).

### Epididymal fat pad

In the 8 week experiment, there was an overall effect of SD photoperiod to decrease epididymal white adipose tissue (EWAT) mass, but no effect of activity or interaction between photoperiod and activity (right fat pad mass only LD-RW 477±58 mg, LD-C 525±27 mg, SD-RW 350±64 mg, SD-C 240±26 mg; F(1,20) = 19.133; P<0.001, F(1,20) = 0.433, P = 0.518, F(1,20) = 2.843, P = 0.107 respectively). Post-hoc analysis shows the only significant difference between individual groups at this time point is between LD-C and the SD-C groups (P<0.001). Consistent with the PTW, EWAT of the SD-RW group was intermediate between the LD-C and SD-C groups. In the 16 week experiment, there was an effect of photoperiod, activity and interaction between photoperiod and activity (LD-RW 431±37 mg, LD-C 382±45 mg, SD-RW 355±20 mg, SD-C 161±10 mg; F(1,27) = 33.16, P<0.001, F(1,27) = 29.04, P<0.001, F(1,27) = 13.246, P = 0.001, respectively). Multiple pair-wise comparison revealed that the significant differences between groups were the SD-C group had reduced EWAT compared to SD-RW (P<0.001) and the LD-C group (P<0.001).

### Liver glycogen and fat

**Figure 4:**
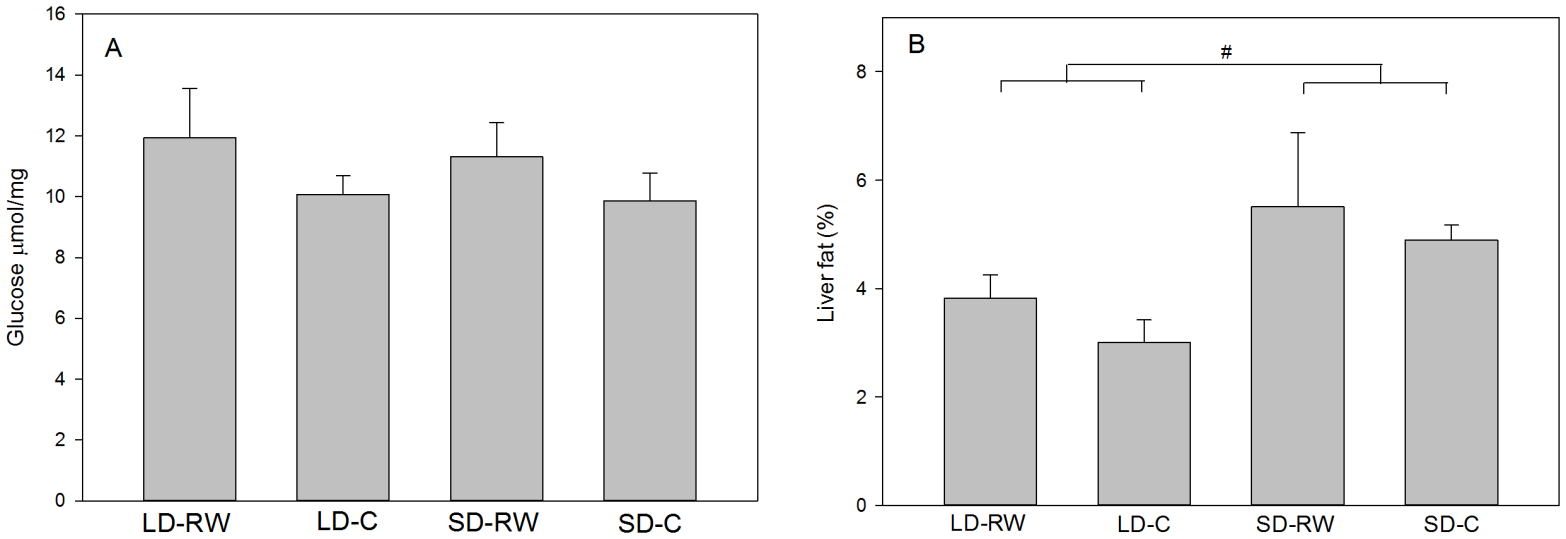
Liver glucose and fat content of adult male Siberian hamsters after 8 weeks exposure to short day (SD) photoperiod or long day (LD) photoperiod or with (RW) or without (C) a running wheel (n = 6 in each group). Results show means ± SEM (#P<0.05, with respect to an overall effect of photoperiod).

To assess whether reduced liver weights in SD are linked to a lowered glycogen and/or fat content, glycogen (as free glucose) and fat were determined. Neither photoperiod or activity had a significant effect on glycogen content in the liver and there was no interaction between photoperiod and activity (F(1,20) = 1.636, P = 0.215; F(1,20) = 3.247, P = 0.087, F(1,20) = 0.087, P = 0.771, respectively; Figure 4A). However, there was a modest effect of photoperiod on liver fat content (F(1,20) = 5.5005; P = 0.029) with a higher percentage of fat in SD compared to LD groups (Figure 4B), but there was no effect of activity or an interaction between photoperiod and activity (F(1,20) = 0.887, P = 0.358, F(1,20) = 0.018, P = 0.893, respectively).

### Photoperiod regulated gene expression in the hypothalamus

#### Genes involved in thyroid hormone regulation and transport

Due to the transient nature of *Dio3* mRNA expression following transfer of hamsters from LD to SD, components of the hypothalamic thyroid hormone regulatory systems (*Dio2*, *Dio3*, T3/T4 transporter *Mct8* and the regulatory thyroid releasing hormone (*Trh*) mRNA were quantified by *in situ* hybridization in the hypothalamus at the end of an 8 week treatment at around the time of expected peak of *Dio3* mRNA expression [4].

**Figure 5:**
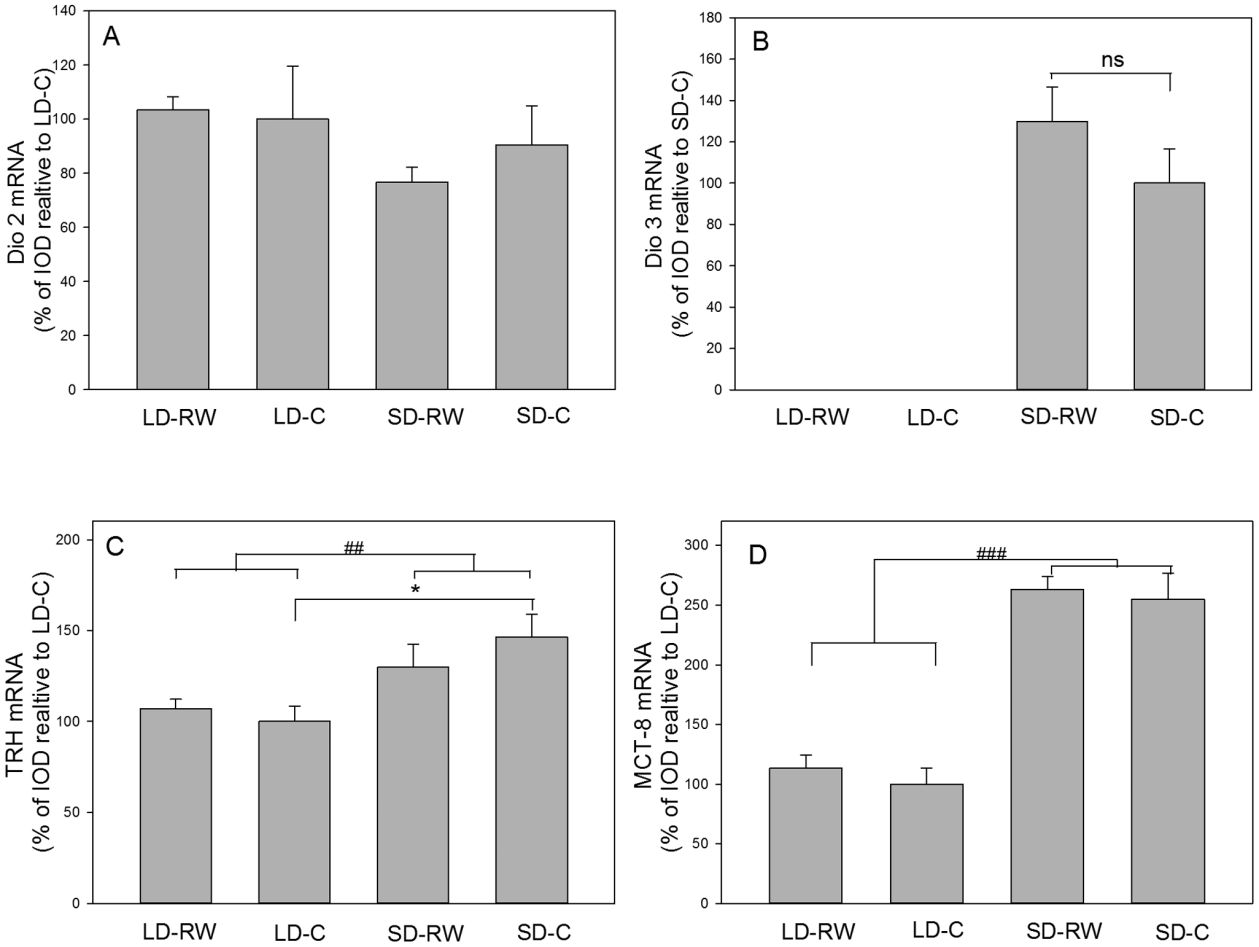
Messenger RNA expression of genes involved in regulation and transport of thyroid hormone. Quantification of (A) type 2 (*Dio2*) and (B) type 3 deiodinase (*Dio3*) mRNA expression in the 3rd ventricular tanycyte layer of adult Siberian hamsters (C) thyroid releasing hormone (*Trh*) mRNA expression in the paraventricular nucleus (PVN) and (D) monocarboxylate transporter 8 (*Mct8*), Hamsters were kept 8 weeks in long day (LD) photoperiod or short day (SD) photoperiod or with (RW) or without (C) a running wheel (n = 6 in each group except Dio2 where n = 5–7 per group). Results shown are means+SEM. The LD-C group was set to 100% expression value (except for Dio3, SD-C set at 100%) and other treatment values were calculated accordingly. Relevant significant differences are shown (###P<0.001, ##P<0.01 with respect to overall difference between photoperiod; *<P0.05 with respect to post-hoc analysis of differences between groups; ns not significant).

*Dio2* gene expression did not differ between any of the groups (Kruskalmc P>0.05 for all comparisons, Figure 5A). *Dio3* mRNA expression was detectable only in the SD groups (Figure 5B) but there was no effect of activity on *Dio3* mRNA expression in SD (P>0.05). Therefore running wheel activity does not affect either *Dio2* or *Dio3* mRNA expression.

Expression of *Trh* mRNA was affected by photoperiod (F(1,20) = 11.57, P = 0.003), but not by activity (F(1,20) = 0.226, P = 0.640) with no interaction between photoperiod and activity (F(1,20) = 1.34, P = 0.261). Post-hoc analysis shows a modest increase in *Trh* mRNA expression in the PVN with expression in the SD-C group being increased by 46% compared to the LD-C group only (P = 0.004; Figure 5C). *Trh* mRNA expression was also measured in a 12 week experiment but there was no difference between the groups after longer SD exposure (Figure S2).

Expression of *Mct8* mRNA was affected by photoperiod (F(1,20) = 105.548; P<0.001) with an increase of 150% in SD compared to LD but was unaffected by running wheel activity (F(1,20) = 0.531; P = 0.475, Figure 5D). No interaction of photoperiod and activity was evident (F(1,20) = 0.035, P = 0.853). SD Mct-8 expression levels were increased relative to the respective LD values in sedentary and exercised hamsters (P<0.001 for both comparisons).

#### Photoperiod genes in the hypothalamic ependymal layer

**Figure 6:**
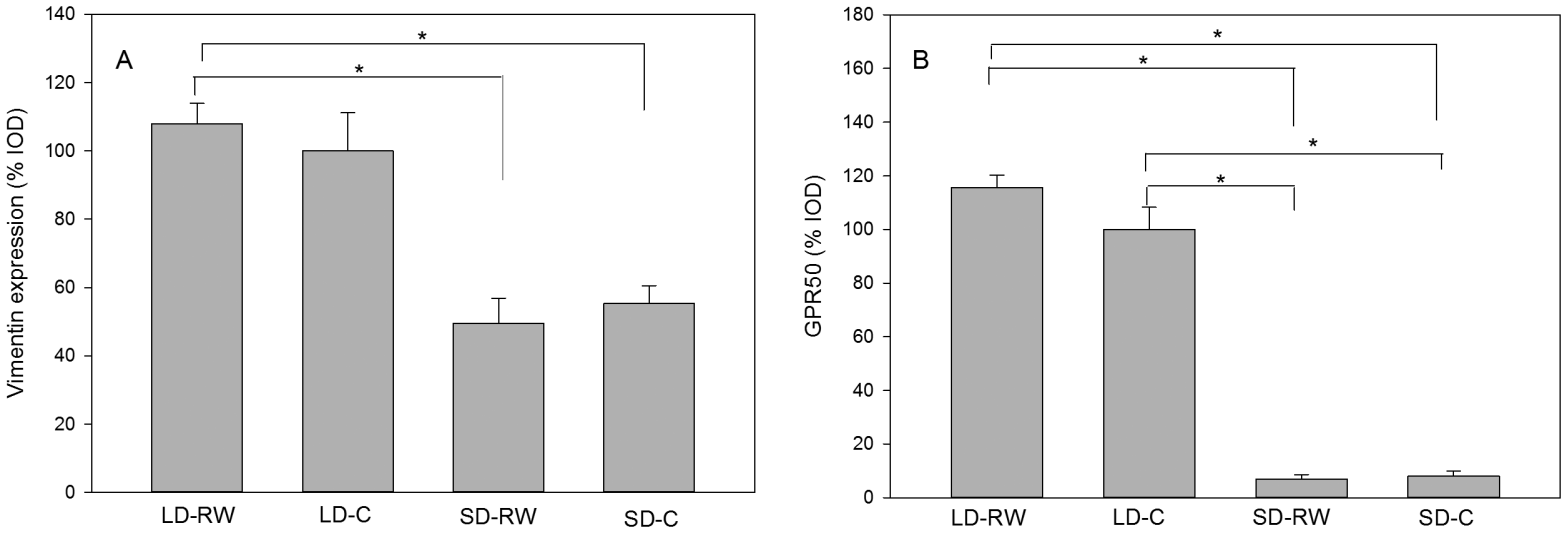
Messenger RNA expression of photoperiod-regulated genes in the ventricular ependymal cells. Quantification of (A) Vimentin and (B) G-protein-coupled receptor 50 (*Gpr50*) mRNA expression. Adult male Siberian hamsters were exposed to long day (LD) photoperiod or short day (SD) photoperiod or with (RW) or without (C) a running wheel for 8 weeks (n = 6–7 in each group). The LD-C group was set to 100% expression value and other treatment values were calculated accordingly. Results show means ± SEM (*P<0.05).

In addition to deiodinase and thyroid hormone transporter regulation, other genes in the ependymal layer are likely to be involved in the contribution of this layer to photoperiod regulated physiology. Examples of ependymal genes under the control of photoperiod include Vimentin, an intermediate filament protein and orphan G-protein-coupled receptor 50 (*Gpr50*). Expression of these genes was quantified in the ependymal layer of the 3^rd^ ventricle in the hamsters from an 8 week experiment. There was an effect of photoperiod on *vimentin* gene expression (Figure 6A) with SD groups showing reduced expression compared to the LD-RW group (Kruskalmc P<0.05), but did not quite achieve significance for the LD-C group. However, there was no effect of running wheel activity on expression levels within photoperiod.

There was an effect of photoperiod treatment on *Gpr50* mRNA expression with higher mRNA expression in LD hamsters (Kruskalmc P<0.05 Figure 6B), but no effect of activity within photoperiod. Both SD groups had reduced *Gpr50* mRNA expression compared to both LD groups (P<0.05)

### Gene expression in the ARC

#### VGF gene expression in the dmpARC

**Figure 7:**
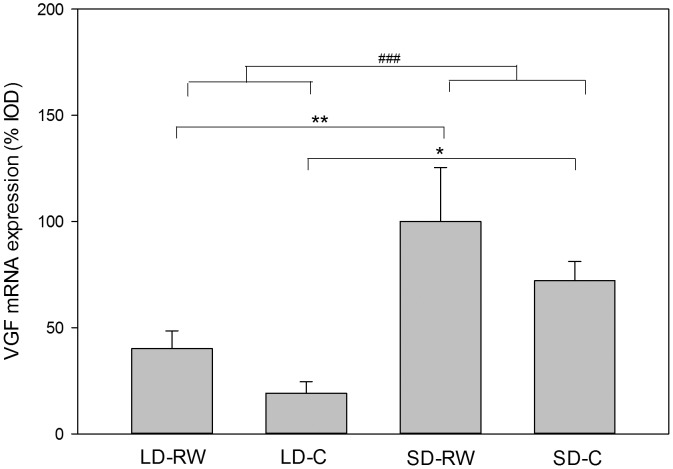
Quantification of *Vgf* mRNA expression in the hypothalamic dorsal medial posterior ARC (dmpARC). Adult male Siberian hamsters were exposed to long day (LD) photoperiod or short day (SD) photoperiod or with (RW) or without (C) a running wheel access for 8 weeks (n = 4–6 in each group). The SD-RW group was set to 100% expression value and other treatment values were calculated accordingly. Results show means+SEM (###P<0.001 with respect to overall difference between photoperiod; ** P<0.01 and *, P<0.05 with respect to post-hoc analysis of differences between groups).

After 8 weeks of treatment, there was an effect of photoperiod on Vgf expression with an increase in the dmpARC in both SD groups (F(1,15) = 20.383; P<0.001, Figure 7). Running wheel activity modestly increased *Vgf* mRNA expression (F(1,15) = 4.655; P = 0.048) but there was no interaction (F1,15) = 0.382, P = 0.546). Within groups, VGF mRNA expression in the LD-RW group was significantly less than SD-RW (P = 0.003) and LD-C less than SD-C (P = 0.013).

#### Orexigenic/anorexigenic gene expression in the ARC

**Figure 8:**
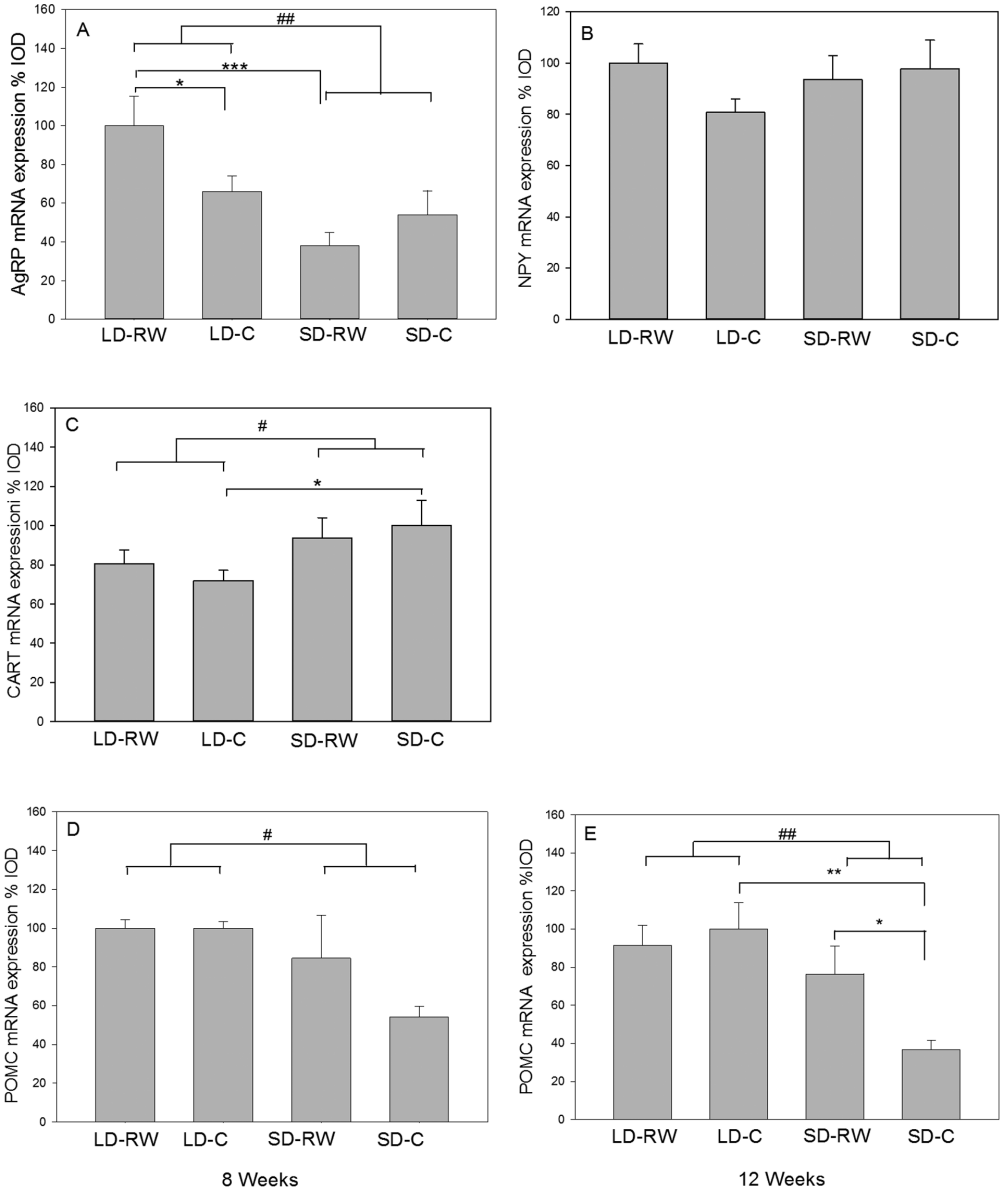
Messenger RNA expression of genes involved in homeostatic mechanisms of appetite and energy balance. Quantification of (A) agouti-related protein (A*grp*), (B) neuropeptide Y (N*py*), (C) cocaine- and amphetamine-regulated transcript (C*art*) and (D) proopiomelanocortin (P*omc*) mRNA expression in the hypothalamic arcuate nucleus (ARC) of adult male Siberian hamsters after 8 weeks exposure to long day (LD) photoperiod or short day (SD) photoperiod with (RW) or without (C) a running wheel (N = 6 in each group). (E) Proopiomelanocortin (*Pomc*) mRNA expression in the hypothalamic arcuate nucleus (ARC) after 12 weeks exposure to long day (LD) photoperiod or short day (SD) photoperiod or with (RW) or without (C) a running wheel (n = 5–6 in each group). The LD-C group was set to 100% expression value and other treatment values were calculated accordingly. Results show means+SEM (##P<0.01, #P<0.05 with respect to an overall effect of photoperiod; *P<0.05, **P<0.01, ***P<0.001, with respect to post-hoc analysis of differences between groups).

Expression of genes for neuropeptides involved in the homeostatic regulation of food intake in the ARC, Agrp, Npy, Cart and Pomc was assessed by in situ hybridization. Two-way ANOVA revealed an effect of photoperiod treatment on Agrp mRNA expression (F(1,20) = 11.025; P = 0.003) after 8 weeks, but no effect of activity (F(1,20) = 0.661, P = 0.426), with an interaction between photoperiod and activity (F(1,20) = 5.041, P = 0.036). Within the LD groups, mRNA expression in LD-RW group hamsters was significantly increased compared to LD-C group (P = 0.043; Figure 8A). Expression was also higher in the LD-RW compared to SD-RW group (P<0.001). However, comparison of LD-C and SD-C groups revealed no effect of photoperiod.

Neither photoperiod nor running wheel activity affected *Npy* gene expression after 8 weeks (Figure 8B) and similar results were found after 12 weeks (Figure S3). *Cart* mRNA expression showed a small but significant effect of photoperiod with higher values in SD after 8 weeks (F(1,20) = 4.76; P = 0.041) as well as the 12 week experiment (Figure S3). However, there was no effect of activity (F(1,20) = 0.017, P = 0.897) or an interaction between photoperiod and activity (F(1,20) = 0.655, P = 0.428). Only within sedentary control groups after 8 weeks, LD-C differed significantly from SD-C (P = 0.047, Figure 8C).

Photoperiod affected *Pomc* gene expression after 8 weeks (F(1,17) = 11.63; P = 0.003). Post-hoc analysis revealed this was attributable to the SD-C group being significantly different compared to LD-C (P = 0.012; Figure 8D). However, there was no effect of activity (F(1,17) = 1.51, P = 0.236) and no interaction between photoperiod and activity (F(1,17) = 1.589, P = 0.224). At 8 weeks, the expression of *Pomc* mRNA in SD-RW group showed an intermediate value between SD-C and LD groups. After 12 weeks there was an overall effect of photoperiod on *Pomc* gene expression (F(1,19) = 10.35, P = 0.005), but no overall effect of activity (F(1,19) = 1.636, P = 0.216) and a trend towards interaction between photoperiod and activity (F(1,19) = 3.928, P = 0.062). Post-hoc analysis reveals the SD-C group decreased *Pomc* mRNA expression compared to LD-C group (P = 0.002;) and the SD-RW group (P = 0.036).

#### *Srif* gene expression in the ARC

**Figure 9:**
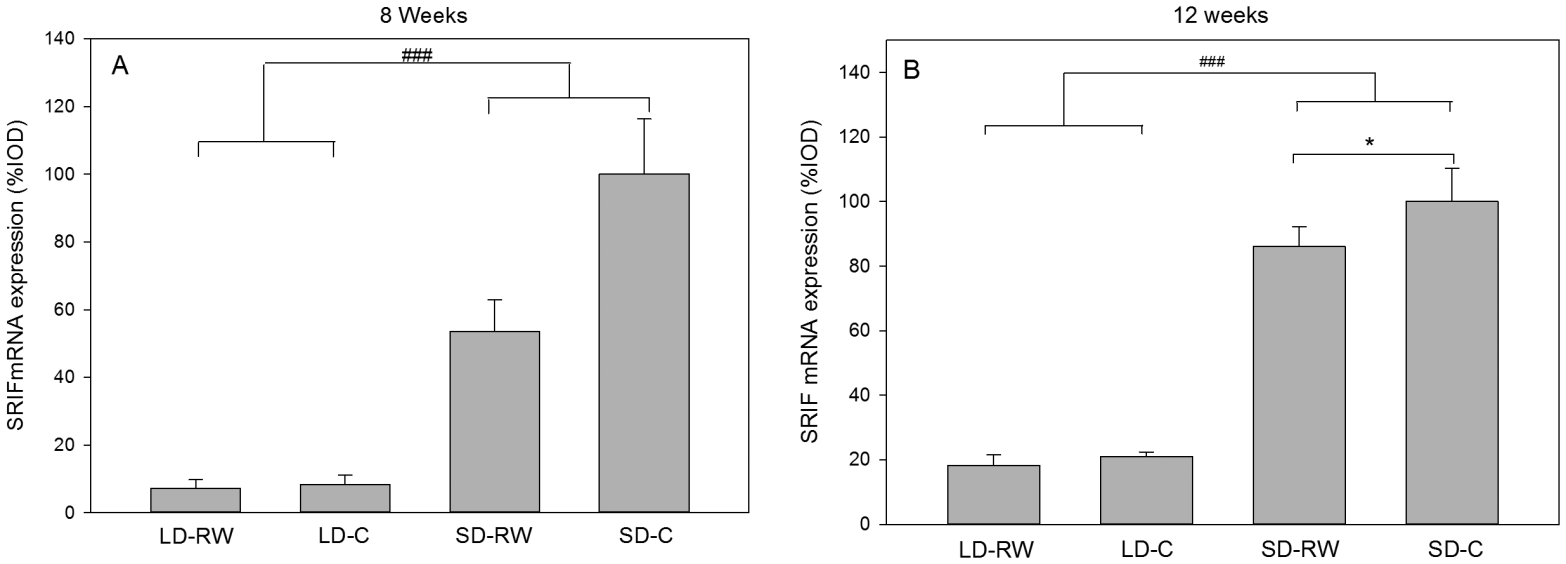
Quantification of somatotropin release-inhibiting factor (*Srif*) mRNA expression in the hypothalamic arcuate nucleus (ARC). Adult male Siberian hamsters were exposed to long day (LD) or short day (SD) photoperiod or with (RW) or without (C) a running wheel for 8 weeks (n = 5–6 in each group). Values are means ± SEM. The SD-C group was set to 100% expression value and other treatment values were calculated accordingly ( ###P<0.001 with respect to an overall effect of photoperiod; *; P<0.05 with respect to post-hoc analysis between groups).

Somatostatin (*Srif*) gene expression in the ARC was analysed as a potential mediator for growth hormone regulation. After 8 weeks, there was an effect of photoperiod (F(1,19) = 77.65, P<0.001), no effect of activity (F(1,19) = 1.703, P = 0.207) and no interaction between photoperiod and activity (F(1,19) = 1.107, P = 0.306). No significant difference was seen between LD groups, but both SD treatments resulted in increased expression of *Srif* mRNA compared to LD groups P<0.001). There was a reduction in expression in the SD-RW compared to the SD-C group, but this did not achieve significance (P = 0.12, Figure 9A). After 12 weeks of treatment the effect of photoperiod was evident (F(1,20) = 115.9, P<0.001) but no effect of activity (F(1,20) = 3.904, P = 0.062) or an interaction between photoperiod and activity (F(1,20) = 2.404, P = 0.137). At this time point there was a small significant difference between SD-C and SD-RW group (P = 0.022, Figure 9B).

### Effect of running wheel activity on peripheral hormones

**Figure 10:**
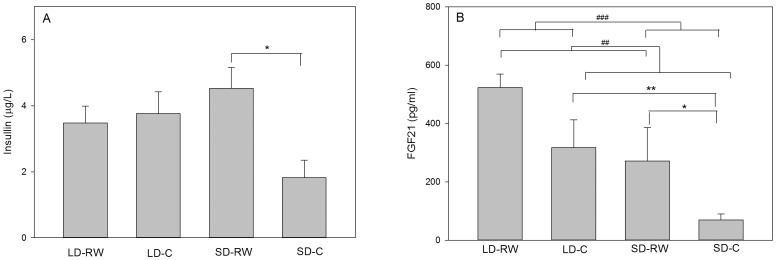
Effect of photoperiod and running wheel activity on circulating concentrations of (A) insulin, (B) FGF21 in adult male Siberian hamsters maintained in long days (LD) or short days (SD) with (RW) or without a (C) running wheel for 12 weeks (LD-RW n = 10, LD-C n = 10, SD-RW n = 9, SD-C n = 8). Values are the mean+SEM (###P<0.001, ##P<0.01 with respect to overall effect of photoperiod or activity; *P<0.05, **P<0.01 with respect to post-hoc analysis between groups).

Circulating insulin was determined in a 12 week running wheel experiment (Figure 10A). More than half of the SD-RW samples exceeded the upper limit of the assay, and for samples exceeding this limit, the upper limit concentration (5.768 µg/L) was used. There was a significantly greater concentration of circulating insulin in the SD-RW running wheel group compared to the SD-C group (Kruskalmc P<0.05), but both LD groups did not achieve significance from the SD-C group in this experiment.

Circulating FGF21 levels (Figure 10B) were significantly different with photoperiod (F(1,33) = 19.4, P<0.001) and activity (F(1,33) = 10.4, P = 0.003), but no interaction (F(1,33) = 1.78, P = 0.191). Post-hoc analysis reveals higher FGF21 values in LD-C over SD-C (P = 0.002). Within SD, SD-RW group have higher values than SD-C (P = 0.02); within LD, LD-RW FGF21 values were not significantly higher than LD-C (P = 0.511) or the SD-RW group (P = 0.831), but were significantly higher than SD-C group (P<0.001).

A summary of the effect of running wheel activity on the SD induced changes in body weight, organ weights, tissue masses and hypothalamic gene expression is presented in Table 2.

**Table 2:** Summary of the physiological, metabolic and hypothalamic gene expression changes in exercising Siberian hamsters with free access to a running wheel compared to sedentary Siberian hamsters in short photoperiod.

	Effect of running wheel activity in short photoperiod		
	8 Weeks	12 Weeks	16 Weeks
Body weight	↑a,c		↑b,c
Fat	↔c		↑b,c
Lean mass	↑a,b,c		↑b,c
Accumulative food intake		↑b	
Serum hormones			
Insulin		↑	
FGF21		↑a,b	
Organ weights			
Liver	↑ b,c		↑a,b
Kidney	↑a,b,c		↑a,b,c
Heart	↔		↑b
EWAT	↔a		↑a,b,c
Testes	↔		↔
Liver Glycogen	↔		
Liver lipid	↔a		
Hypothalamic expression of mRNA			
AgRP	↔a,c		
NPY	↔	↔	
POMC	↔a	↑a	
CART	↔a	↑a	
SRIF	↔a	↑a	
Dio2	↔	↓a	
Dio3	↔		
TRH	↔a	↔	
MCT-8	↔a		
Vimentin	↔		
GPR50	↔		
VGF	↔a,b		

↑↓ indicates a significant elevated or decreased value, ↔ indicates no change or the change is not significant. The suffix a, b or c indicates an overall significant effect (P<0.05 or less) for photoperiod (a), activity (b) or interaction between photoperiod and activity (c).

## Discussion

In the current studies, as noted previously, running wheel activity prevented body weight loss in SD hamsters when access to a running wheel was permitted from the onset of SD photoperiod [31]. The mechanistic basis of the effect of exercise on physiology is unknown, but we hypothesised that the effect may be due to a reversal of key gene expression changes in the hypothalamus of the Siberian hamster involved in photoperiod-induced adaptation of body weight physiology.

MRI scanning of decapitated bodies was used to assess body composition of the Siberian hamster. Although values obtained are not absolute, the data show very good correlation between lean mass or fat mass and body weight and provides relative values for comparison. When this analysis is applied to sedentary and running wheel hamsters it becomes evident that there is no reduction in lean and fat mass at either 8 or 16 weeks of exposure to SD in hamsters with a running wheel whereas this was evident in SD sedentary hamsters. Whilst these experiments were performed on hamsters given access to a running wheel at the beginning of the experimental photoperiod exposure, body weight can also increase following a photoperiod induced decrease in body weight [30]. This finding would suggest that the underlying drive to seasonal body weight physiology is overridden.

Contributing to this difference of effect on body mass, the liver, kidney and fat masses were maintained at LD values in hamsters exposed to SD and with access to a running wheel. Paired testes weight was significantly heavier at both 8 and 16 weeks in the LD groups compared to SD-C group, while the SD-RW group at an intermediate value was not significantly different from the other three groups. Together this indicates that the underlying photoperiod drive is present, but that activity provides a hormonal input to the testis which prevents full regression. Prevention of testicular regression, induction of testicular growth or induction of estrous cyclicity in anestrous females has also been shown in exercised Syrian hamsters [41], [42], [43].

The maintenance or accretion of fat mass is a significant feature of the effect of running wheel activity. This increase in lean and fat mass by activity occurs in spite of an increase in energy expenditure. When measured as accumulated food intake over 12 weeks, we found an increase in total food intake which by the end of 12 weeks was greater in hamsters with running wheels than the sedentary groups. This higher intake in RW hamsters likely compensates for the increased energy expenditure due to the increase in activity and/or anabolic effects (i.e. growth). The reduction in organ weights in SD compared to LD hamsters has previously been noted [31], but the question remains as to whether this is due to a change in tissue mass or other factors such as glycogen or fat content. However, analysis of liver glycogen and fat content did not reveal any differences which could account for either the effect of photoperiod or that of exercise. Therefore we conclude the difference in liver weight is due to tissue mass.

The most parsimonious explanation for the observed changes in body mass is a reversal of photoperiod-driven changes in hypothalamic gene expression involved in seasonal adaptations of body weight. A key element of the adaptive mechanism involved in seasonal physiological response is the reduction of hypothalamic thyroid hormone (T3) availability [4], [44]. In the Siberian hamster, this occurs as a result of T3 catabolism by an increase in Dio3 deiodinase in tanycytes of the hypothalamic ventricular layer and a small reduction in Dio2 deiodinase, the anabolic enzyme for T3 production located in the same cells during SD exposure [4], [8], [21]. An optimal time in the photoperiodic response for *Dio3* mRNA expression is at 8 weeks of SD exposure [4]. Analysis of *Dio2* and *Dio3* mRNA expression by *in situ* hybridisation after 8 weeks of treatment in the current studies revealed that these pivotal enzymes involved in the photoperiodic response are not altered by wheel running activity. In addition, photoperiod regulation of expression of the T3 transporter MCT-8 was also unaffected. These data together suggest that wheel running activity does not act via the hypothalamic thyroid hormone system to increase body weight in the Siberian hamster.

In addition to the regulation of genes involved in thyroid hormone transport and metabolism, an increasing cohort of genes is now known to be regulated by photoperiod in tanycytes [2], [3], [6], [7], [24]. Two additional genes were chosen to assess whether exercise could impact on other aspects of tanycyte physiology: Vimentin is an intermediate filament protein with a possible role in morphological adaption of tanycytes at the neuroendocrine interface between the hypothalamus and median eminence; GPR50 is an orphan G protein-coupled receptor for which there is evidence for a role in energy metabolism and a gatekeeper function for torpor induction in hamsters and mice [45]–[47]. Both these genes are down-regulated in SD, however, exercise had no effect on expression of these genes either in SD or LD. These data show that exercise does not have a generalised impact on photoperiod-regulated gene expression in tanycytes and therefore on tanycyte physiology.

With no apparent effect of exercise on photoperiod regulated gene expression in tanycytes, we next considered whether exercise alters neuronal gene expression. Increased food intake with exercise may be anticipated to be reflected in genes involved in the regulation of the homeostasis of energy balance. However, no effect was observed on orexigenic *Npy* and *Agrp* gene expression that would account for increased food intake. There is a small overall increase in anorexigenic *Cart* gene expression in SD as previously noted [48], but this increase was unaffected by exercise. Expression of *Pomc* mRNA was decreased in SD sedentary hamsters in both an 8 week and 12 week experiment compared to the LD sedentary group. This decrease in *Pomc* mRNA expression has previously been observed in SD hamsters and although counterintuitive, may lead to an increase in the anorexigenic peptide, α-MSH [16]. There was no effect of exercise in LD, but SD-RW hamsters had an intermediate level of expression between LD and SD sedentary hamsters at 8 weeks. The SD-RW group was not different from the LD-C or LD-RW group at 12 weeks, but different from the SD-C group. An effect of exercise on *Pomc* mRNA expression is not without precedent as increases have been observed in diet-induced obese rats subject to exercise [49].

Our previous studies have identified an increasing cohort of photoperiod-regulated genes in the area of the hypothalamus termed the dmpARC. The dmpARC is electrophysiologically activated in SD due to the decrease in histamine H3 receptor expression and in a region in which an increase in VGF mRNA expression occurs. A peptide derived from the pro-peptide (TLQP) is able to reduce food intake and body weight in the Siberian hamster [50]. Consequently, VGF was chosen as a representative of a photoperiod-regulated gene for the dmpARC. However, no effect of exercise was found in either LD or SD hamsters. As an output of an activated region in SD, it seems likely that upstream effects i.e. on H3 receptor and *c-fos* mRNA expression for example, would be unaffected by exercise.

Recently, we and others have reported that *Srif* (somatostatin) mRNA expression shows a large increase in the ARC of SD hamsters [13], [51], [52]. The significance of this change is not yet known but could be an integral part of a growth response mechanism with an anticipated inhibitory effect on the growth hormone axis in SD. After 8 weeks, there was an increase in *Srif* mRNA expression in SD sedentary hamsters. This increase was evident in SD hamsters with access to a running wheel, although there was an indication that the increase found in sedentary hamsters was blunted by exercise. The involvement of the growth hormone axis through regulation of somatostatin or growth hormone by photoperiod or exercise would not be without precedent as evidence of involvement of this axis has been noted in the Syrian hamster [43], [53]–[55]. The present data leads us to the conclusion that exercise does not alter whole-scale photoperiod-induced gene expression to increase body weight of SD hamsters. These findings would be consistent with the observation that when given access to a running wheel in SD for several weeks leading to an increase in body weight, body weight will decrease quickly when the running wheels are locked [31]. This implies that the SD melatonin signalling driving photoperiod changes in the pars tuberalis and downstream via tanycytes and neuronal mechanisms remains intact and able to bring about the photoperiod-programmed weight loss. Further evidence of an uninterrupted melatonin signal is the maintenance of pelage colouration toward winter colouration in SD hamsters despite running wheel activity [31].

Two fundamental questions remain to be answered in this model: What is the mechanism by which exercise increases body weight and why does this occur?

As with the realisation that adipose tissue is an endocrine organ, we now appreciate that muscle is also an endocrine organ that potentially secretes a large array of proteins termed myokines [56]–[58]. Hence we hypothesize that running wheel activity of the Siberian hamster leads to the release of myokines that feedback to a central mechanism to increase food intake, and through a central mechanism, peripheral mechanism or a combination of both to bring about an increase in body weight.

Insulin is a peripheral hormone with a lipogenic potential in the Siberian hamster [26] and has previously been found to circulate at higher concentrations in LD hamsters compared to SD [25], [27, Barrett et al unpublished data]. Analysis of circulating insulin concentrations showed a higher mean value of insulin in the LD-C compared to SD-C, but did not achieve significance in this experiment. This is due to individual variability in circulating insulin which may be due to several factors such as the insulin being measured in the non-fasted state. However, insulin levels were significantly greater in exercising SD-RW hamsters compared to SD-C implicating peripheral insulin resistance and a mechanism for the maintenance of fat deposition. This is contrary to the effect of exercise in other rodent models or humans where exercise lowers insulin, increases insulin sensitivity and enhances the central effect of insulin to reduce food intake [59].

FGF21 is an insulin stimulated myokine whose concentration in circulation increases with exercise [60], [61]. Serum samples from a 12 week photoperiod/running wheel experiment revealed an overall higher level of FGF21 in LD compared to SD and an increase due to running wheel activity. In a recent study, exogenous FGF21 was shown to induce body weight loss in Siberian hamsters and the study noted a higher value for endogenous circulating FGF21 in SD compared to LD [27]. These contrasting results can be explained by the imposition of a 16 h fasting period in the previous study, a treatment that would be expected to increase FGF21 which, in conjunction with an already reduced body fat content in SD hamster, could lead to a more acute response of FGF21. FGF21 has been shown to blunt the lipolytic action of growth hormone [62]. Therefore elevated levels of FGF21 as a result of running wheel activity would be consistent with our hypothesis that the maintenance of fat with running wheel activity is partly attributable to the inhibitory effect of FGF21 on lipolysis and partly attributable to the lipogenic effect of insulin in adipocytes [26]. However, in respect of both insulin and FGF21 we can conclude that exercise maintained the hormonal status of LD.

In conclusion, reversal or prevention of short day body weight loss in the Siberian hamster by running wheel activity is achieved through maintenance of both fat mass and lean tissue including most organ masses. The mechanism underlying the response to exercise does not however, involve a reversal of short day induced hypothalamic gene expression changes that are known, or likely to contribute to body weight loss, but exercise overrides the photoperiod induced mechanism for body weight reduction. Further work is necessary to identify the underlying molecular basis for this response.

## Supporting Information

Figure S1 **Correlation of fat and lean mass in exercised and sedentary Siberian hamsters.** A scatter plot of lean or fat mass as determined by nuclear magnetic resonance imaging analysis vs body weight for (A) Siberian hamsters held in photoperiod for 8 weeks or (B) 16 weeks. (n = 6–9 per group). Closed circles; Long day with running wheel; Open circles; Long day sedentary; Closed triangles; Short day with running wheels; Open triangle; Short day sedentary.
(TIF)

Figure S2 **12 week photoperiod treatment -** ***Trh*** **mRNA expression.** Quantification of *Trh* mRNA expression in the PVN of Siberian hamsters in long days (LD) or short days (SD) with (RW) or without (C) a running wheel. The duration of photoperiod exposure was 12 weeks (n = 5–6 per group). LD-C group value was set to 100% and other groups adjusted accordingly. There were no significant differences between treatments at this time point.
(TIF)

Figure S3 ***Npy*** **and** ***Cart*** **mRNA expression at 12 weeks of photoperiod treatment.** Quantification of (A) *Npy* and (B) *Cart* mRNA expression in the ARC of Siberian hamsters in long days (LD) or short days (SD) with (RW) or without (C) a running wheel. The duration of photoperiod exposure was 12 weeks (n = 6 per group). LD-C group value was set to 100% and other groups adjusted accordingly. *P<0.05 between LD and SD groups.
(TIF)

Table S1 ***In situ*** **hybridization probes.** Accession numbers and regions used for the design of PCR primers to amplify DNA sequences for use as templates to generate antisense or sense probes for *in situ* hybridization.
(DOCX)
