# Supplementary figures and images for: Effect of Exercise on Photoperiod-Regulated Hypothalamic Gene Expression and Peripheral Hormones in the Seasonal Dwarf Hamster Phodopus sungorus
Source: PLoS One. 2014 Mar 6;9(3):e90253. doi: 10.1371/journal.pone.0090253 (PMC3946023; doi:10.1371/journal.pone.0090253)

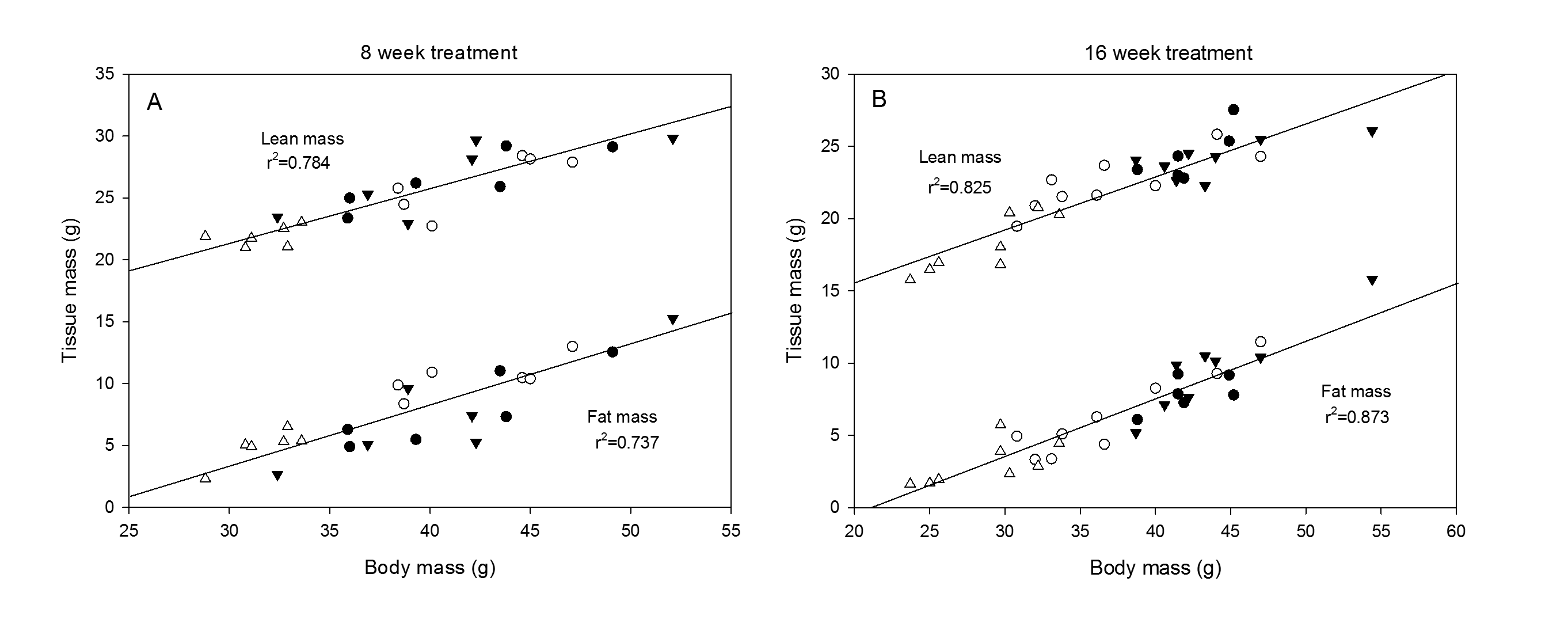

Supplement: Figure S1 — Correlation of fat and lean mass in exercised and sedentary Siberian hamsters. A scatter plot of lean or fat mass as determined by nuclear magnetic resonance imaging analysis vs body weight for (A) Siberian hamsters held in photoperiod for 8 weeks or (B) 16 weeks. (n = 6–9 per group). Closed circles; Long day with running wheel; Open circles; Long day sedentary; Closed triangles; Short day with running wheels; Open triangle; Short day sedentary. (TIF) [file pone.0090253.s001.tif]

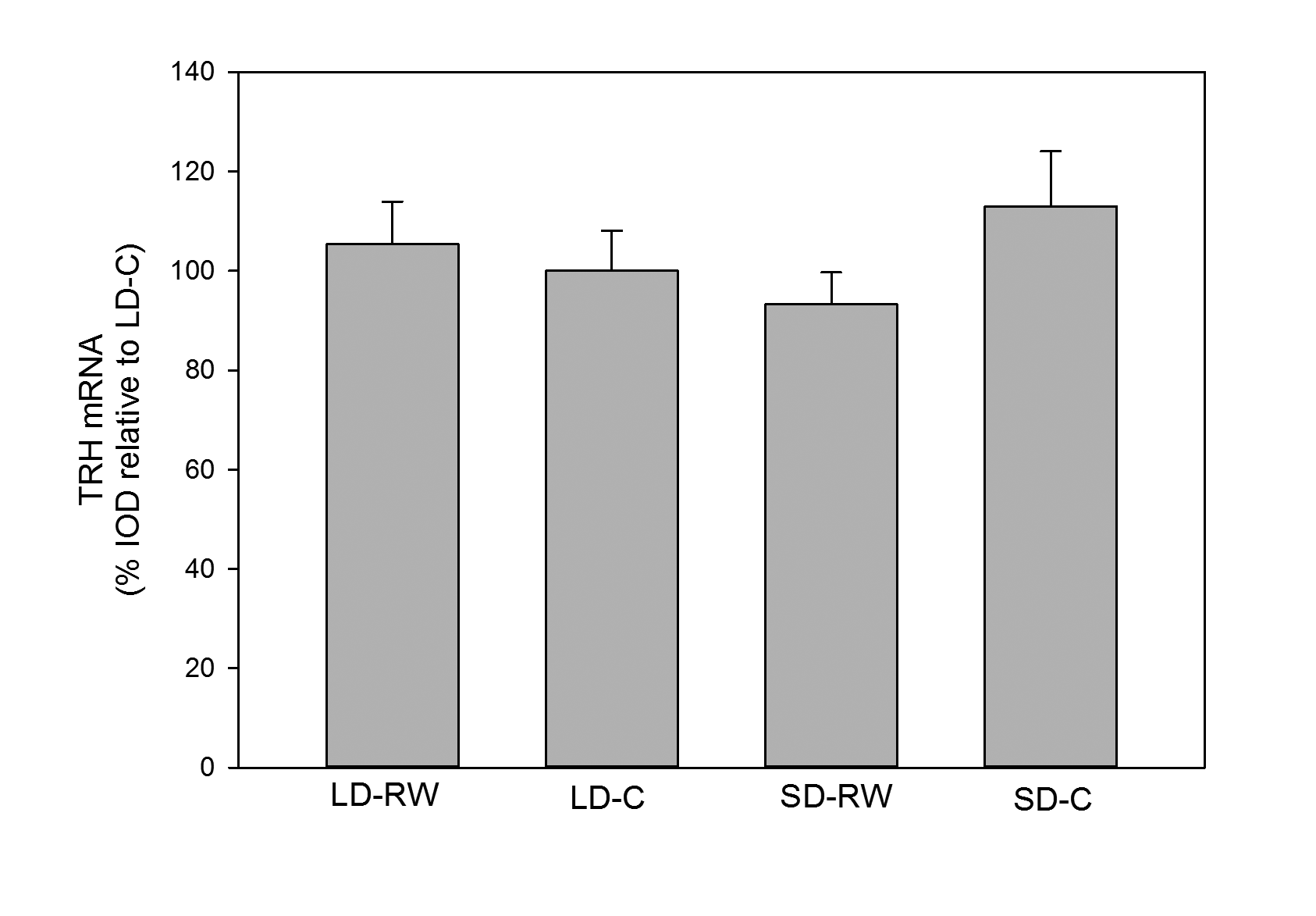

Supplement: Figure S2 — 12 week photoperiod treatment - Trh mRNA expression. Quantification of Trh mRNA expression in the PVN of Siberian hamsters in long days (LD) or short days (SD) with (RW) or without (C) a running wheel. The duration of photoperiod exposure was 12 weeks (n = 5–6 per group). LD-C group value was set to 100% and other groups adjusted accordingly. There were no significant differences between treatments at this time point. (TIF) [file pone.0090253.s002.tif]

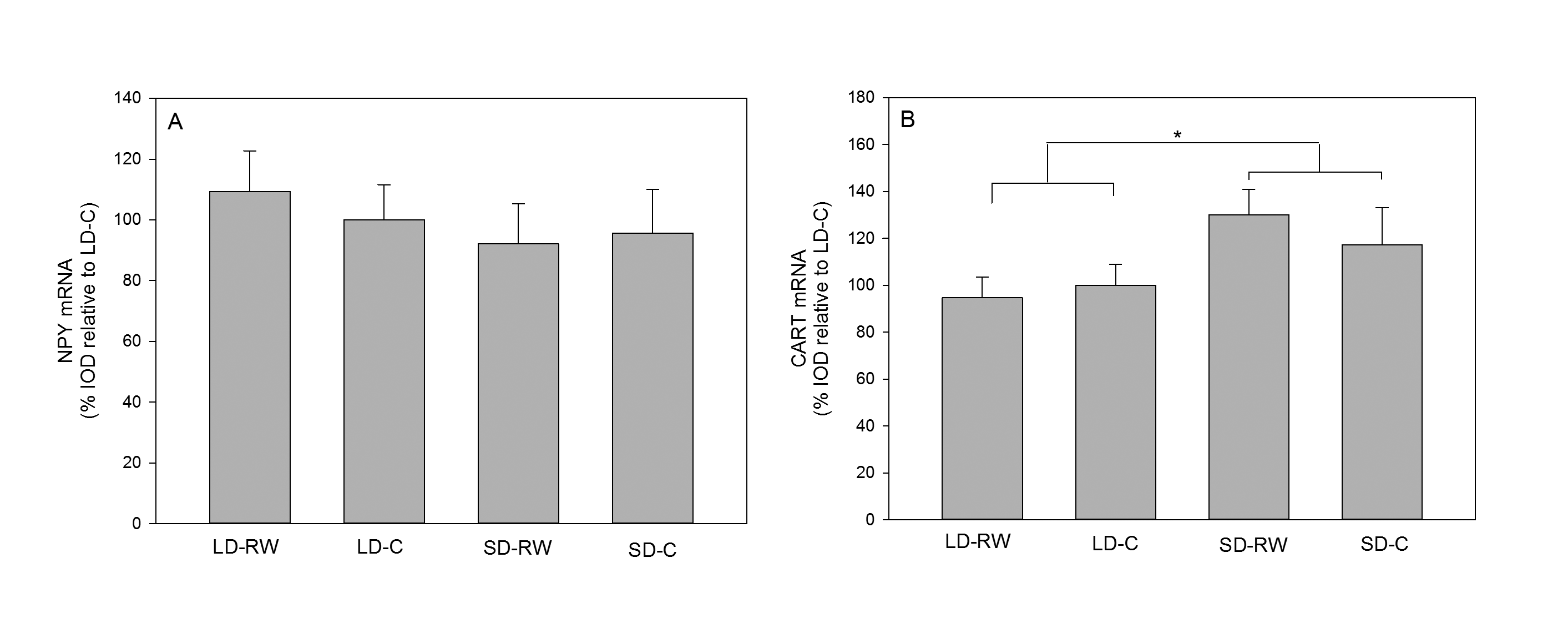

Supplement: Figure S3 — Npy and Cart mRNA expression at 12 weeks of photoperiod treatment. Quantification of (A) Npy and (B) Cart mRNA expression in the ARC of Siberian hamsters in long days (LD) or short days (SD) with (RW) or without (C) a running wheel. The duration of photoperiod exposure was 12 weeks (n = 6 per group). LD-C group value was set to 100% and other groups adjusted accordingly. *P<0.05 between LD and SD groups. (TIF) [file pone.0090253.s003.tif]
