# Supplementary material for: Effect of Exercise on Photoperiod-Regulated Hypothalamic Gene Expression and Peripheral Hormones in the Seasonal Dwarf Hamster Phodopus sungorus
Source: PLoS One. 2014 Mar 6;9(3):e90253. doi: 10.1371/journal.pone.0090253 (PMC3946023; doi:10.1371/journal.pone.0090253)
Supplement: Table S1 — In situ hybridization probes. Accession numbers and regions used for the design of PCR primers to amplify DNA sequences for use as templates to generate antisense or sense probes for in situ hybridization. (DOCX) [file pone.0090253.s004.docx]

| Gene | Accession number | In situ hybridization probe region |
| --- | --- | --- |
| Dio2 | AF096875 | 67-560 |
| Dio3 | NM_001010993 | 16-405 |
| Vimentin | NM_011701 | 780-1291 |
| Srif | NM_012659 | 202-499 |
| Trh | M12138 | 125-355 |
| Gpr50 | AF065145 | 206-451 |
| Vgf | M60525 | 801-1573 |
| Mct8 | NM_147216 | 351-866 |
| Cart | U10071 | 26-363 |
| AgRP | U89484 | 113-341 |
| Pomc | J00162 | 263-665 |
| Npy | M20373 | 1-511 |
